# Using developmental psychotechnics during COVID-19 pandemic: The case of children and adolescents failing to follow covid-related guidelines

**DOI:** 10.1192/j.eurpsy.2021.819

**Published:** 2021-08-13

**Authors:** N. Burlakova, V. Oleshkevich

**Affiliations:** 1 Department Of Neuro- And Pathopsychology, Lomonosov Moscow State University, Moscow, Russian Federation; 2 Academic Department, Scientific-Practical Children’s Mental Health Centre n. a. G. Sukhareva of Moscow City Department of Healthcare, Moscow, Russian Federation

**Keywords:** psychotechnics, applied clinical psychology, COVID-19

## Abstract

**Introduction:**

The problem of COVID-19 is acute now in all the countries. Nevertheless, the techniques of applied clinical psychology are rarely implemented in this struggle.

**Objectives:**

The aim was to examine the possibilities of the applied clinical psychology in instructing children and adolescents and in exerting psychological influence on them in order to decrease the incidence.

**Methods:**

The following methods were used: thematic analysis of relevant information on TV and on Russian-speaking internet, interviews with adolescents refusing to wear masks (110 adolescents), observation.

**Results:**

87% of adolescents are sure that they are “fully informed about the COVID-19.” Moreover, 70% are certain they “will not be infected with COVID-19” and even if they do, they will “have a mild form of the disease.” Such information was widely reproduced in the Russian media and proved by medical statistics. It influenced negatively the attitude of adolescents toward masks, social distancing, etc.

**Conclusions:**

1) The information concerning COVID-19 requires introduction of the changes based on psychological data. 2) The ways of informing children and adolescents should be carefully analyzed using psychological data. In information for young people, their responsibility, solidarity and empathy toward others should be involved. 3) The situation of COVID-19 may become a space for potential development, this situation may shape respect toward the health of other people. 4) The “situation of test,” which is significant for the self-awareness of adolescents, should be employed as a way to persuade adolescents to follow the health restrictions.

